# Co-production of bioethanol and probiotic yeast biomass from agricultural feedstock: application of the rural biorefinery concept

**DOI:** 10.1186/s13568-014-0064-5

**Published:** 2014-09-01

**Authors:** Claire M Hull, E. Joel Loveridge, Iain S Donnison, Diane E Kelly, Steven L Kelly

**Affiliations:** 1 Institute of Life Science, College of Medicine, Swansea University, Swansea SA2 8PP, Wales, UK; 2 Institute of Biological, Environmental & Rural Sciences, Aberystwyth University, Gogerddan, Aberystwyth SY23 3EE, Wales, UK

**Keywords:** Bioethanol, Biomass, Biorefinery, Cholesterol, Probiotic, Saccharomyces boulardii

## Abstract

Microbial biotechnology and biotransformations promise to diversify the scope of the biorefinery approach for the production of high-value products and biofuels from industrial, rural and municipal waste feedstocks. In addition to bio-based chemicals and metabolites, microbial biomass itself constitutes an obvious but overlooked by-product of existing biofermentation systems which warrants fuller attention. The probiotic yeast *Saccharomyces boulardii* is used to treat gastrointestinal disorders and marketed as a human health supplement. Despite its relatedness to *S. cerevisiae* that is employed widely in biotechnology, food and biofuel industries, the alternative applications of *S. boulardii* are not well studied. Using a biorefinery approach, we compared the bioethanol and biomass yields attainable from agriculturally-sourced grass juice using probiotic *S. boulardii* (strain MYA-769) and a commercial *S. cerevisiae* brewing strain (Turbo yeast). Maximum product yields for MYA-769 (39.18 [±2.42] mg ethanol mL^−1^ and 4.96 [±0.15] g dry weight L^−1^) compared closely to those of Turbo (37.43 [±1.99] mg mL^−1^ and 4.78 [±0.10] g L^−1^, respectively). Co-production, marketing and/or on-site utilisation of probiotic yeast biomass as a direct-fed microbial to improve livestock health represents a novel and viable prospect for rural biorefineries. Given emergent evidence to suggest that dietary yeast supplementations might also mitigate ruminant enteric methane emissions, the administration of probiotic yeast biomass could also offer an economically feasible way of reducing atmospheric CH_4_.

## Introduction

There is global impetus towards development of biorefineries that utilise industrial, rural and municipal waste for the production of bioenergy and marketable bio-based compounds. The biorefinery concept has been identified as a significant opportunity for rural economic development (Charlton et al. [2009]; Leistritz and Hodur [2008]; Sharma et al. [2011]) and perennial ryegrass (*Lolium perenne* L.) is currently under investigation as a non-food crop that could be processed as feedstock in a rural biorefinery setting (Farrar et al. [2012]). Production of biogas from ryegrass pulp (Kyazze et al. [2008]) and bioethanol from grass juice (Martel et al. [2010]; Martel et al. [2011]) has already been achieved. Microbial biotechnology and metabolic engineering promises to diversify the application of the biorefinery approach for production of novel products and several ‘designer yeast strains’ capable of using polyfructose have been reported (Martel et al. [2011]; Wang et al. [2011]; Zhang et al. [2010]). Nonetheless, yeast species that already have GRAS (generally regarded as safe) status remain readily applicable to biorefinery processes and novel uses for yeast biomass warrant consideration. In the present study, we investigate the co-production of bioethanol and probiotic yeast biomass from enzyme-pretreated grass juice (Martel et al. [2010]).

In addition to its use in fermentation, food and biofuel industries, the brewing yeast *Saccharomyces cerevisiae* has several health applications. It is used as a protein supplement, immune enhancer and is employed as a vehicle for the introduction of dietary compounds as a commercialised health product (Moyad [2008]). The yeast *S. boulardii* is administered for the treatment of gastrointestinal disorders (Buts [2009]; Vandenplas et al. [2009]; Zanello et al. [2009]) and is currently the only commercially available probiotic yeast. The ability of *S. boulardii* to ferment ethanol has been documented (Gurgu et al. [2011]) as have certain physiological and growth characteristics (Edwards-Ingram et al. [2007]) including evidence that it can assimilate cholesterol (Chen et al. [2010]; Psomas et al. [2003]). However, despite its genetic relatedness to *S. cerevisiae* (Edwards-Ingram et al. [2004]) and use as a human probiotic for over 50 years, the alternative applications of *S. boulardii* are not well studied. Given growing interest in the biotherapeutic properties of different yeasts (Foligne et al. [2010]) there is now a clear incentive to develop and apply research knowledge about food grade yeasts.

The purpose of this study was to investigate if the co-production of bioethanol and probiotic yeast biomass is a feasible strategy for enhancing the productivity and value of rural biorefineries of the future. We sought to determine the potential bioethanol and yeast biomass yields attainable from agriculturally-sourced grass juice using *S. boulardii* (MYA-769) and a commercial *S. cerevisiae* brewing strain (Turbo yeast). Both strains of yeast are safe and the methodology reported in the present study (from feedstock extraction to product utilisation) compatible with land availability, rural land use patterns, current legislation and the existing technology base in the United Kingdom (Charlton et al. [2009]; Farrar et al. [2012]; Martel et al. [2010]). The applications of yeast biomass as a feed additive and/or probiotic for livestock in the rural biorefinery setting are discussed.

## Materials and methods

### Yeast strains and growth media

Bioethanol and biomass co-production studies were undertaken using a commercial brewing strain of *Saccharomyces cerevisiae* (Turbo yeast; Gert Strand AB) and a probiotic strain of *Saccharomyces boulardii* (MYA-769; ATCC). Both were maintained at 30°C on yeast-peptone-dextrose (YPD) medium containing (w/v): 2% glucose, 2% bacto peptone and 1% yeast extract (±2% agar). All media components were supplied by Difco^TM^ (BBL/Difco Laboratories). All other chemicals were supplied by Sigma (Sigma-Aldrich Ltd) unless otherwise stated.

Grass juice (GJ) feedstock was extracted from ryegrass *Lolium perenne* supplied by the Institute of Biological, Environmental and Rural Sciences (IBERS, UK) (Martel et al. [2010]). GJ was screened to remove large particulates, autoclaved (121°C, 30 min) and frozen (-80°C). When required for use as a growth and fermentation substrate, particle-free GJ was thawed and component fructans enzymatically hydrolysed using truncated *L. paracasei* β fructosidase (_t_fosEp) as previously described (Martel et al. [2010]). The concentration of free monosaccharides in untreated and enzyme pre-treated GJ + _t_fosEp was determined and the latter chosen for use as feedstock for all experimental work undertaken in the present study.

For sterol assimilation studies, cholesterol-supplemented glucose yeast minimal media (_glc_YM^+chol^) containing 1.34% yeast nitrogen base without amino acids, 2% glucose and 10 μg mL^−1^ cholesterol (final concentration) was prepared. Cholesterol was dissolved in 1:1 Tween 80:ethanol to give a 2 mg mL ^-1^ stock and filter-sterilised prior to use.

### Sugar assay

Sugar analyses were performed on 2500-fold diluted GJ and GJ + _t_fosEp in 100 mM potassium phosphate, pH 7.0, containing 10 mM MgSO_4_, 1 mM NAD^+^, 1.5 mM ATP and 20 U mL^−1^*Leuconostoc mesenteroides* glucose-6-phosphate dehydrogenase (Worthington Biochemical Corporation). Concentrations of glucose, fructose, sucrose and fructan were determined from the changes in absorbance at 340 nm following sequential addition of 20 U mL^−1^ *S. cerevisiae* hexokinase (Worthington Biochemical Corporation), 20 U mL^−1^*E. coli* phosphoglucose isomerase (Megazyme International Ireland Ltd), 1.5 U mL^−1^ *S. cerevisiae* sucrase/maltase (Megazyme International Ireland Ltd) and 150 μg _t_fosEp (purified as previously described Martel et al. [2010]) respectively. Standards of glucose, fructose, sucrose and chicory inulin were used to calibrate the assay.

### Growth and fermentation studies

Growth and fermentation experiments were performed in 100-well honeycomb microplates using a Bioscreen C (Oy Growth Curves Ab Ltd, Finland). Uniform starting (t_0_h) culture densities were achieved by resuspending individual yeast colonies in GJ and diluting to obtain 5 × 10^5^ cells mL^−1^ in GJ. Starting cultures were vortexed and aliquotted into Bioscreen wells (300 μL volumes). All experiments were incubated at 20°C for optimal bioethanol production as previously described (Martel et al. [2011]) and optical density readings (at 600 nm) taken every 45 min. Data was exported from the Bioscreen in ASCII format prior to analysis using Excel (Microsoft Office 2003).

Growth parameters were derived using standard methodology. Briefly, ΔOD values describe maximum OD – minimum OD; the lag phase is defined as the length of time a culture spends at < 10% of maximum OD; T_½_Max values are equivalent to the time taken to reach half the maximum increase in growth of a culture (ΔOD × 0.5). Fastest doubling times (DT) were estimated by dividing the natural logarithm of 2 by the fastest culture growth rates (μ), where μ is the gradient of the linear trend line fitted to log-transformed OD data.

Screening of experimental cultures for bacterial contamination and observations of the cell morphology of both yeast strains were made using a Nikon Eclipse E600 microscope.

### Bioethanol and biomass

At specific time intervals (t_0_h, t_24_h, t_48_h, t_72_h, t_96_h t_100_h and t_124_h) Bioscreen measurements were suspended and a 10 μL volume of culture supernatant removed from representative experimental wells. These 10 μL volumes were immediately diluted (10-, 100- and 1000-fold) with distilled water and frozen for subsequent ethanol analysis. Ethanol determinations were made using a spectrophotometric assay kit (K-ETOH 11/06; Megazyme Ltd) according to manufacturer’s instructions. For biomass yield estimations (g dry weight L^−1^), the contents of 10 unsampled Bioscreen wells were pooled at t_124_h and dried to constant mass using a centrifugal evaporator (Heto Maxi Dry Plus).

### Cholesterol assimilation experiments

Individual colonies from Turbo yeast (*S. cerevisiae*) and MYA-796 (*S. boulardii*) agar plates were used to inoculate 10 mL volumes of _glc_YM^+chol^ media. These starter cultures (3 replicates per yeast species) were maintained at 37°C for 48 h in static (no agitation) 30 mL sterilin vials to attain low-oxygen conditions. At t_48_h, cell pellets were harvested by centrifugation and washed three times with sterile water prior to sterol extraction, derivatisation and analysis using gas chromatography-mass spectrometry (GC-MS).

### GC-MS sterol analysis

Washed cell pellets from cultures grown using YPD, GJ + _t_fosEp and _glc_YM^+chol^ were resuspended in 7:3 methanol:water containing 18% (w/v) potassium hydroxide and 0.1% (w/v) pyrogallol and heated at 90°C for 2 h. Non-saponifiable sterols were extracted into glass HPLC vials using 3 × 2 mL volumes of hexane. Extracts were evaporated to dryness using a centrifugal evaporator (Heto Maxi Dry Plus) and derivatised using 100 μL N,O-bis(trimethylsilyl)trifluoroacetamide and trimethylchlorosilane (BSTFA-TMCS [99:1]) and 50 μL anhydrous pyridine at 70°C for 2 h.

Trimethylsilyl (TMS)-derivatised sterols were analyzed using a 7890A GC-MS system (Agilent Technologies) with a DB-5MS fused silica column (30 m × 0.25 mm × 0.25 μm film thickness; J&W Scientific). The oven temperature was initially held at 70°C for 4 min, then increased at 25°C min^−1^ to a final temperature of 280°C, which was held for a further 25 min. Samples were analyzed in splitless mode (1 μL injection volume) using helium carrier gas, electron impact ionization (ion source temperature of 150°C) and scanning from m/z 40 to 850. GC-MS data files were analysed using MSD Enhanced ChemStation software (Agilent Technologies) to determine sterol profiles for all isolates and for derivation of integrated peak areas. Sterols were identified by reference to retention times and mass fragmentation patterns for known standards.

## Results

Results from the present study demonstrate the potential to co-produce bioethanol and probiotic yeast biomass from grass juice feedstock and identify avenues for process development and application in rural birefinery settings.

**Figure 1:**
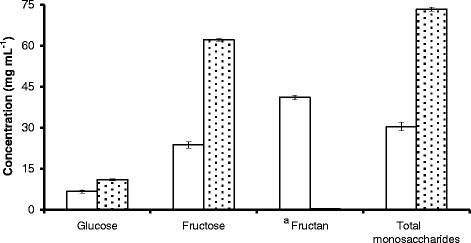
**Mean [±S.D] glucose, fructose and fructan concentrations in untreated GJ (open bars) and GJ** + _**t**_**fosEp (filled bars).**^a^ Fructan = polyfructose.

Pre-treatment of grass juice (GJ) feedstock with the soluble, truncated core domain of *Lactobacillus paracasei* β-fructosidase (_t_fosEp) purified from recombinant *Escherichia coli* (Martel et al. [2010]) resulted in the complete hydrolysis of non-fermentable fructan moieties (Figure 1). The total monosaccharide (glucose and fructose) content of GJ + _t_fosEp (73.31 [±0.67] mg mL^−1^) was over two-fold higher than that of untreated GJ (30.39 [±1.51] mg mL^−1^); the sucrose content of both was negligible (0.60 [±0.06] mg mL^−1^). Grass juice contains smaller amounts of other sugars (e.g., galactooligosaccharides and maltosaccharides) in addition to proteins which can also be used for fermentation and growth; non-fermentable carbohydrates (i.e., lignin, cellulose and hemicellulose) are found in grass pulp and the fibrous biomass fraction (Charlton et al. [2009]). GJ + _t_fosEp was found to support optimal yeast growth, bioethanol and biomass production and was consequently employed as the feedstock for all experimental work reported in the present study.

**Figure 2:**
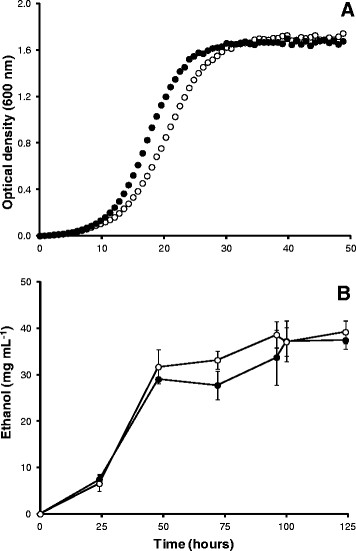
**Growth (A) and bioethanol fermentation (B; mean values [±S.D]) of turbo yeast (●) and MYA-796 (○) grown using GJ+**_**t**_**fosEp.** Note that ethanol concentrations were sampled after yeast cultures had reached stationary phase (typically t_48_h); ethanol concentrations decreased after t_125_h.

The growth parameters determined for *S. cerevisiae* (Turbo yeast) and *S. boulardii* (MYA-769) were closely comparable (Table 1 and Figure 2) indicating that the composition of GJ + _t_fosEp did not inhibit the growth of either yeast. The maximum bioethanol and biomass product yields for Turbo (37.43 [±1.99] mg ethanol mL^−1^ and 4.78 [±0.10] g dry weight L^−1^) and MYA-769 (39.18 [±2.42] mg ethanol mL^−1^ and 4.96 [±0.15] g dry weight L^−1^) were also very similar.

**Table 1:** **Mean [±S.D] growth parameters for Turbo yeast and MYA-796 grown on enzyme-pretreated grass juice (GJ** + _**t**_**fosEp)**

	**Growth parameters**				**Maximum product yield**	
	Δ**OD**_**600**_	**Lag (h)**	**T**_**½**_**Max (h)**	**Max DT (h)**	**Bioethanol (mg mL**^**−1**^**)**	**Biomass (g L**^**−1**^**)**
Turbo	1.70 [0.02]	11.25 [0.5]	17.25 [0.5]	3.5 [0.5]	37.43 [1.99]	4.78 [0.10]
MYA-796	1.74 [0.02]	12.00 [0.5]	20.25 [0.5]	4.5 [0.5]	39.18 [2.42]	4.96 [0.15]

ΔOD_600_ = maximum – minimum optical density reading (at 600 nm); Lag phase = length of time population remains at < 10% of maximum OD; T_½_Max **=** time taken to achieve half maximal population growth (maximum OD – minimum OD × 0.5); Max DT = maximum observed doubling time.

**Figure 3:**
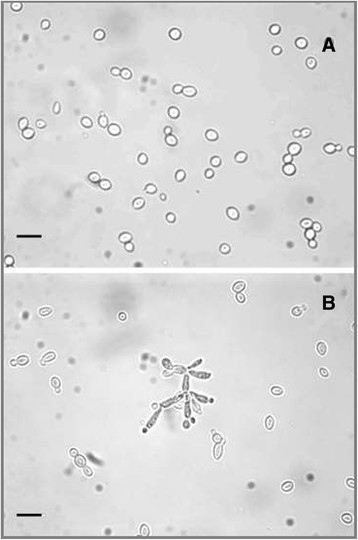
**Cell morphology of yeast strains. A** = yeast-like growth of turbo (*S. cerevisiae*) showing solitary blastoconidia and normal budding; **B** = pseudohyphal growth of MYA-796 (*S. boulardii*). Scale bars represent 15 μm.

Microscope observations revealed that the two yeasts exhibited different growth morphologies (Figure 3). Turbo cultures were characterised by round solitary blastoconidia and normal cellular budding (Figure 3A) while MYA-769 cultures comprised a mixture of yeast-like, elongated and psuedohyphal growth forms (Figure 3B).

**Figure 4:**
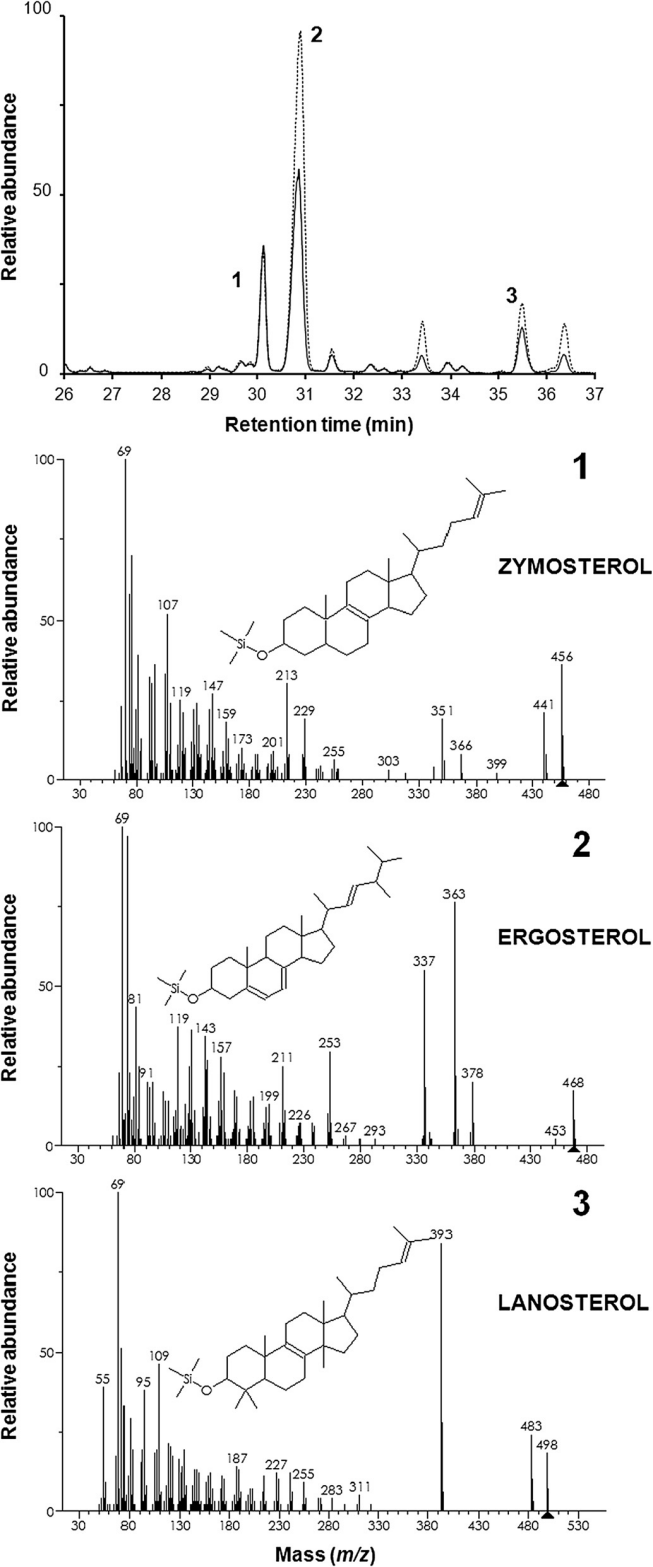
**Sterol composition.** Overlay of GC-MS sterol chromatograms for turbo yeast (unbroken trace) and MYA-796 (broken trace) grown on GJ + _t_fosEp. Diagnostic fragmentation spectra for **1**) zymosterol, **2**) ergosterol and **3**) lanosterol are shown; note the presence of minor sterol intermediates (retention times 31.5-34.5 min and 36.5 min).

**Figure 5:**
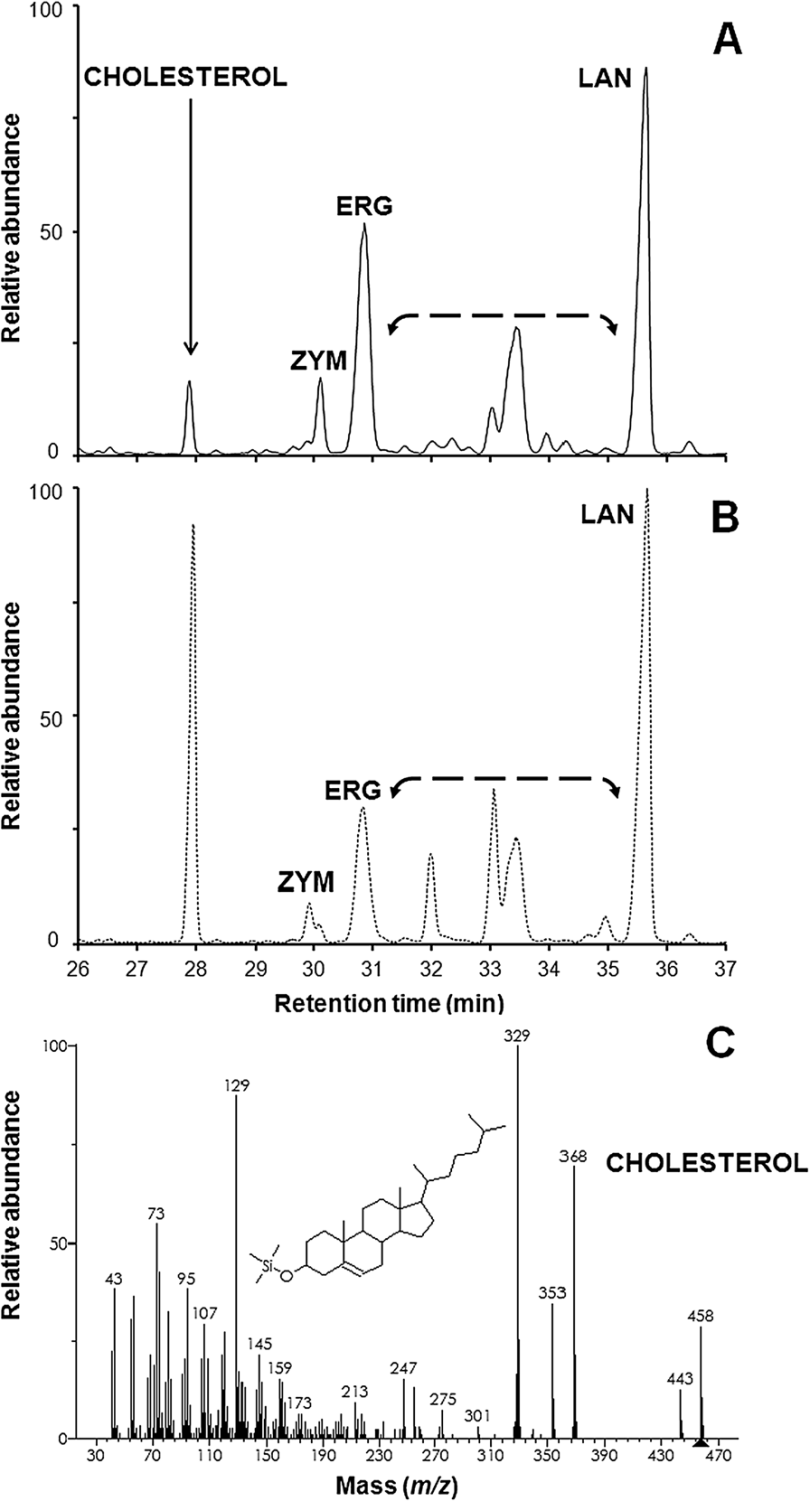
**Cholesterol assimilation experiments.** Overlay of GC-MS sterol chromatograms for **A)** turbo yeast (unbroken trace) and **B)** MYA-796 (broken trace) grown on _glc_YM^+chol^. The diagnostic fragmentation spectrum for cholesterol is shown **(C)**. Note the increased abundance of minor sterol intermediates (bracketed arrow) relative to cultures grown on GJ + _t_fosEp and YPD media.

Results from cholesterol uptake experiments (Table 2 and Figures 4 and 5) indicate that, under oxygen-limited conditions and at a growth temperature compatible with that of the human body (37°C) MYA-769 assimilated more cholesterol than Turbo (Table 2 and Figure 5).

**Table 2:** Mean [±S.D] cellular sterol composition (%) of turbo yeast and MYA-796

	**Sterol composition (%)**									
	**Zymosterol**		**Ergosterol**		**Lanosterol**		^**a**^**Intermediates**		**Cholesterol**	
	**Turbo**	**MYA-769**	**Turbo**	**MYA-769**	**Turbo**	**MYA-769**	**Turbo**	**MYA-769**	**Turbo**	**MYA-769**
**YPD**	17.92 [0.38]	17.79 [0.49]	**61.71 [3.29]**	**61.91 [1.39]**	5.93 [1.65]	8.06 [0.59]	14.45 [2.02]	12.25 [1.49]	—	—
**GJ** + _**t**_**fosEp**	15.53 [2.80]	12.31 [0.10]	**67.31 [0.49]**	**61.86 [1.10]**	5.90 [0.09]	9.31 [0.76]	11.26 [3.20]	16.52 [0.23]	—	—
_**glc**_**YM**^**+chol**^	5.7 [0.78]	1.6 [0.41]	26.9 [1.27]	12.2 [2.12]	**35.7 [0.74]**	**38.2 [3.64]**	27.10 [1.79]	27.30 [2.05]	4.6 [0.48]^**b**^	20.7 [0.96]^**b**^

The most abundant sterol in each experiment is emboldened. ^a^ = sum of all minor sterol intermediates (each comprising < 5% of total cellular sterol fraction); ^b^ = exogenously supplied cholesterol; Strikethrough = not detected.

## Discussion

### Bioethanol and biomass

*S. cerevisiae* has traditionally been used in food production, biotechnology, brewing and biofuel industries; however, the bioethanol and biomass production observed in the present study highlights the potential to utilise *S. boulardii* for industrial ethanol fermentation processes (Gurgu et al. [2011]). That GJ + _t_fosEp is a suitable feedstock for yeast biofermentations is evidenced by the sterol composition of both yeast species following growth on standard YPD media and GJ + _t_fosEp (Table 2 and Figure 4). Neither yeast was affected by perturbations in ergosterol biosynthesis; ergosterol is an essential yeast membrane sterol needed to maintain membrane viability and healthy growth (Daum et al. [1998]). Morphological differences were observed when Turbo and MYA-769 were grown using both GJ + _t_fosEp and standard YPD media suggesting that the psuedohyphal growth of MYA-769 (Figure 3) is a typical strain characteristic and not a response to nutrient limitation; nutrient limitation is understood to be a prerequisite for filamentous growth in wild-type *S. cerevisiae* (Gimeno et al. [1992]). *S. boulardii* is reported to possess an enhanced ability for pseudohyphal switching and is understood to survive better at low pH than other strains of *S. cerevisiae* (Edwards-Ingram et al. [2007]).

Given these morphological and physiological characteristics, *S. boulardii* presents itself as an attractive microorganism for biotechnology and industrial applications where flocculent and sedimenting strains of *S. cerevisiae* are particularly valuable (Kida et al. [1990]; Seong et al. [2006]). A flocculation mutant of *Candida glabrata*, another species showing genetic relatedness to *S. cerevisiae* (Roetzer et al. [2011]), has been identified as a potentially useful strain for bioethanol production because of its growth at higher temperatures (Watanabe et al. [2009]).

### Cholesterol assimilation

Research and commercial interest surrounds the biotherapeutic properties of different yeasts (Foligne et al. [2010]) and those with cholesterol-lowering activity have attracted specific attention (Chen et al. [2010]; Psomas et al. [2003]). *S. cerevisiae* is already known to sequester extracellular cholesterol under anaerobic conditions (Lorenz et al. [1986]); however, results from the present study indicate cholesterol uptake by *S. boulardii* (strain MYA-769) is superior. In view of the impetus towards lowering cholesterol through dietary modifications and speculation that probiotic yeast could provide a means to lower serum cholesterol (Chen et al. [2010]; Krasowska et al. [2007]) work to characterise cholesterol uptake in the host environment using a wider number of strains is now required. Phytosterols were not detectable in the sterol chromatograms for Turbo yeast or MYA-796 grown using GJ + _t_fosEp (Table 2 and Figure 4), and indeed the sterol content of GJ was negligible; however, we did find residual ryegrass pulp, a by-product of the grass juice extraction process, to be rich in plant sterols (data not shown). The potential to extract phytosterols from ryegrass and alternative plant biomass feedstock requires consideration not least because diet supplementations containing plant sterols could offer protection against a variety of chronic ailments including cardiovascular diseases, obesity, diabetes, and cancer (Bradford and Awad [2007]).

### Applications for yeast biomass

Given legislation restricting the addition of antibiotics to animal feed (Seo et al., [2010]) the potential to market food-grade yeast biomass and/or utilise it in a rural biorefinery setting is important. The use of *S. boulardii* for treatment of human gastrointestinal disorders is well documented (Buts [2009]; Vandenplas et al. [2009]; Zanello et al. [2009]) and studies indicate that probiotic yeast could also be administered as a direct-fed microbial to improve livestock quality (Collier et al. [2010]; Keyser et al. [2007]). The effects of dietary yeast (*S. cerevisiae*) autolysate on poultry health have also been documented (Yalçin et al. [2012]). Finally, there is emerging evidence to show that dietary yeast supplementations could mitigate ruminant enteric methane emissions (Cottle et al. [2011]; Lila et al*.*[2004]). Methane is the second most important greenhouse gas and considering the importance of ruminant livestock, probiotic administration could offer an economically feasible way of reducing ruminant CH_4_ production while improving productivity (Shibata and Terada [2010]).

In conclusion, rural biorefineries have been identified as a potential means to facilitate social and economic regeneration in regions where a low GDP affects communities (Charlton et al. [2009]). Here we demonstrate the possibility of generating bioethanol and probiotic yeast biomass from agriculturally-sourced grass juice. Breeding for bio-ethanol production using *L. perenne* L. with higher water-soluble carbohydrate content is already underway in the United Kingdom (Farrar et al. [2012]) and results from the present study highlight further opportunities for integrated microbial biotechnology and large scale biorefining using high sugar perennial ryegrasses (Charlton et al. [2009]; Martel et al*.*[2010]; Martel et al. [2011]). The experiments described can be a base towards extraction of maximum value from grass as proteins and chlorophyll are some of the other products that can be envisaged. The grass cellulosic fibre could also be fermented after appropriate enzymological and process treatments. Increased content of water soluble carbohydrates in breeding processes and engineering solutions to concentrate the juice to generate higher concentrations of bioethanol for distillation are also avenues that need to be explored. Finally it is evident *S. boulardii* could be used in other bioethanol processes if a use for biomass on that scale was desirable as in reducing methane emissions.

## Competing interests

The authors declare that they have no competing interests.

## Authors’ contributions

CMH designed and undertook the growth studies and conceived and drafted the manuscript. EJL carried out the sugar assays. ISD and DEK participated in the design of the study and written work. SLK conceived the study, and participated in its design and coordination and helped to draft the manuscript. All authors read and approved the final manuscript.

## Authors’ information

EJL undertook experimental work at Swansea University, his current contact address is now: School of Chemistry, Cardiff University, Main Building, Park Place, Cardiff, CF10 3AT, Wales, UK.
